# Relationship between breakfast consumption, BMI status and physical fitness of Ghanaian school-aged children

**DOI:** 10.1186/s40795-020-00344-9

**Published:** 2020-04-01

**Authors:** Reginald Adjetey Annan, Solomon Adjetey Sowah, Charles Apprey, Nana Ama Frimpomaa Agyapong, Satoru Okonogi, Taro Yamauchi, Takeshi Sakurai

**Affiliations:** 1 Department of Biochemistry and Biotechnology, Kwame Nkrumah University of Science and Technology, Kumasi, Ghana; 2 Faculty of Agriculture, The University of Tokyo, Tokyo, Japan; 3 Faculty of Health Sciences, School of Medicine, Hokkaido University, Sapporo, Japan

**Keywords:** Anthropometry, Breakfast consumption, Physical fitness, Physical activity, School-aged children, Ghana

## Abstract

**Background:**

Good nutrition and physical activity of school-aged children are important for ensuring optimum growth and reducing obesity. This present study assessed associations between breakfast consumption, BMI-for-Age (BMI) and physical fitness in a cross-section of school-aged children attending government-owned primary schools in Kumasi, Ghana.

**Method:**

The sample consisted of 438 pupils (boys = 213; girls = 225; mean age 11.1 ± 1.1), attending 10 randomly selected schools. Weight (kg), height (cm) and Mid Upper Arm Circumference (MUAC) were measured for each participant, and BMI-for-age z-scores determined using the World Health Organisation (WHO) anthroplus software. Participants were stratified into thinness, normal weight, overweight/obese using WHO cut offs. Physical fitness was assessed using forward jump, left and right handgrips, flexibility, sit-ups and 50 metre run following standard procedures and converted to scores of 1 to 10 following Japanese standards, based on which percentiles were derived. Total fitness score for each pupil was computed by adding all scores. A questionnaire was used to assess meal intake patterns.

**Results:**

The mean BMI-for-age z-score for participants was − 0.24 ± 0.99. Thinness, normal weight and overweight/obesity were 2.7, 86.5, and 10.5% respectively among the pupils. Overweight was higher in girls (14.2%) compared to boys (4.2%), *p* = 0.003. Similarly, mean MUAC was significantly (*p* = 0.021) higher in the girls (22.0 ± 3.2 cm) than the boys (20.7 ± 7.3 cm). For physical fitness, the girls scored higher in forward jump (*p* < 0.0001), 50-m run (*p* = 0.002) and overall fitness score than the boys (21.0 ± 6.2 versus 19.2 ± 8.3, *p* = 0.012). However, a larger proportion of boys performed excellently and poorly than girls (*p* = 0.019). A positive correlation was observed between BMI z-score and hand grip (*r* = 0.21, *p* < 0.001), while sit up (*r* = − 0.11, *p* = 0.018) showed a negative correlation with BMI z-score. No other fitness test varied by BMI. Overweight children performed best in handgrip. Majority of children said they engaged in exercise (89.9%) and consumed breakfast (78.9%). Breakfast consumption was not associated with BMI z-score (x^2^ 0.0359, *p* = 0.549) but non-breakfast consumers performed better in 50 m run compared to consumers (7.0 seconds ± 2.3 vrs 6.3 seconds ± 2.5, *p* = 0.022). Children who reported to exercise were physically fitter than those who did not.

**Conclusion:**

Underweight levels were low while overweight was over 10% in these children. Girls were more than 3 times affected by overweight than boys, and were also physically fitter than boys. Breakfast consumption was not related to weight or fitness.

## Background

Good nutrition and physical activity have many health benefits in school-aged children, including ensuring optimum growth and reducing obesity [1]. Conversely, a significant percentage of mortality and morbidity among children globally is attributed to poor nutritional practices; most commonly manifested as undernutrition (stunting and wasting) [2]. The adverse health effects of poor nutritional status and low physical fitness is amplified by their propensity to cluster among school-aged children (6-12 years), presenting a huge health, social and economic burden [3].

Childhood represents a critical period of human development where modifiable health behaviours acquired may be carried on through adolescence and into adulthood [4]. Evidence of poor dietary practices and increased sedentariness among school-aged children in Sub-Saharan Africa have accumulated over the years [5]. The shift from ‘leptogenic’ to obesogenic physical environments, coupled with extensive use of electronic media such as television viewing and playing video games are among the factors mediating the lack of physical activity among children [6]. Also, increasing access to high caloric “westernized” diets at cheaper prices, massive marketing campaigns of high sugar and low nutrient foods by industries have been suggested to underline poor eating habits among children [7]. These can predispose school-aged children to childhood obesity, micronutrient deficiencies, as well as chronic diseases, such as diabetes, cardiovascular diseases and some forms of cancers in adulthood [8]. Compelling evidence has showed a global decline in fitness level of children worldwide [9], an indication of an increased cardiometabolic risk [10]. In developing countries such as Ghana, the consequences are more profound owing to the already weak national health systems [11, 12].

Few studies have been conducted to assess the nutritional status and physical fitness of school-aged children in developing countries [13]. In fact, no study has investigated the physical fitness of Ghanaian school children and how this might be influenced by nutritional status. Such data will immensely aid the design and implementation of evidence-based interventions within this age group. This study was designed to compensate for the inadequacy of data on nutritional status and physical fitness, including their relationship among school-aged children in Ghana.

## Methods

### Design and sample

This was a cross-sectional study. The sample comprised 442 class five pupils (215 boys and 227 girls), aged between 8 and 13 years, from 10 schools randomly selected from government-owned primary schools in the Kumasi Metropolis. Due to missing data, 438 (213 boys; 225 girls) participants were included in the analysis. The survey was approved by the Kumasi Metropolitan Assembly as well as the heads of the selected schools. Informed consents to participate were also sought from parents/guardians of each participating pupil. Children between 9 and 13 years were eligible to participate in the survey. Physically challenged and pupils who could not perform the various tests as per instructions due to physical injuries or illness were excluded. Also, those who refused to partake in the study were not included.

### Data collection

Data were collected between September and October 2016. A total of 6 field assistants underwent 3 days training and one full day trial on the procedures of data collection. Among the data collection team were nutritionists and health scientists. A pilot study was carried out in one school prior to data collection. Pupils provided details of their date of birth, age, as well as parents’ and guardians’ names and occupation. The phone numbers of their parents or guardians were also recorded for follow-up.

### Assessment of underweight and overweight prevalence

All anthropometric measurements were carried out using standardized protocols. Each anthropometric measurement was taken twice and the average recorded. A platform beam scale was used to assess body weight (Tanita Digital Bathroom Scale HD-660-WH). Students were asked to empty their pockets and took off any belt or jewellery. They were then instructed to stand on the scale with their heads up and arms hanging at their sides. The weight was recorded in kilograms once the reading in the scale’s digital readout had stabilized. The heights of the pupils were measured using a SECA stadiometer (CE 0213), set up against a wall. Students stood straight on the footboard with their feet slightly apart and their heels touching the back of the footboard. The movable headpiece was brought down to touch the top of their heads and the measurements were recorded in centimetres. A flexible tape measure was used to measure Mid Upper Arm Circumference (MUAC). The acromion process of the scapula was located with the tip of the tape measure while the arm was positioned at a 90-degree angle. The tape measure was brought to the elbow and the tip of the trochlea located. The length of the arm was then measured and the mid-point of this length determined. A mark was made to indicate this position and the tape measure was wrapped firmly around the arm without compressing the tissue. The MUAC was then recorded in centimetres. Body Mass Index was calculated for each child using the weight and height measures in the formula BMI = weight (kg)/height (m^2^) and the BMI z-scores were computed using the WHO antroplus software. This allowed for stratification of children into four [4] groups; thinness, normal or healthy weight, overweight and obese, however three groups were realized as none of the children fell within the obese category. The WHO recommends using thinness; BMI z-scores to measure undernourishment in children above 5 years old. Thinness is therefore used to describe BMI z-score less than − 2 Standard Deviation (SD), overweight greater than + 1 SD (equivalent to BMI 25 kg/m2 at 19 years) and obesity greater than + 2 SD BMI (equivalent to BMI 30 kg/m2 at 19 years).

### Assessment of physical fitness

The following components of physical fitness were assessed; musculoskeletal component (hand grip strength, forward jump or standing broad jump), flexibility component (flexibility and sit-ups), and the motor component (50 m run) [14] . All the following test measurements were taken twice except for the 50 m run. Demonstrations and explanations were given before the assessment begun and as needed thereafter. A hand grip dynamometer was used to measure handgrip. Pupils were asked to hold the dynamometer in their left hands first and squeezed when told to. This was done for the right hand as well. After each reading, the instrument was set to zero for the next reading. The measurement was recorded in kg. To measure forward jump, children stood at the starting line and were instructed to jump forward as far as possible, taking off with both feet. The distance from the starting line to the heel of the closer foot was recorded. Two trials were recorded to the nearest 1 cm, and the average of the two was recorded for analysis. For sit up measurements, the test was scored as the number of sit-ups performed in 30s. Each child laid down on a mat placed on flat floor, with both knees bent and both hands under the head. The enumerator held the child’s ankles. The participants’ elbows had to touch the ipsilateral knee. After each upward movement, the participants returned to the starting position. The forward flexion of the trunk (flexibility) test was scored as the most distant point reached on a ruler with the fingertips by bending the body at the waist. Both thumbs had to touch each other, and the knees had to be straight. Two trials were recorded to the nearest 0.5 cm, and the average of the two was retained for analysis. For the 50 m run, the children sprinted as fast as possible to the finish line; 50 m from the starting line. Time taken for each child to cross the finish line was recorded to the nearest 0.1 s using stopwatches.

The raw values of the physical fitness tests were converted to standard scores using Japanese physical fitness performance standard for children, based on age and gender. This standard score template consists of a total of eight physical fitness tests scores, ranging from one to ten, for each physical fitness test, with one being the lowest and ten the highest. After the conversions, all the standard scores were added to get a total fitness score, and the fitness level determined for each child as excellent, very good, average or poor, based on age and gender. In this study however, just five of the physical fitness activities were performed by the children. Hence overall fitness score, computed from the score for each activity was based on five rather than eight activities. The scores were used as a continuous variable so that the higher the score, the fitter the child. Also, percentiles were created for the total standard scores because it was impossible to use the classifications created in the Japanese template. Participants who scored above the 75th percentile were classified as excellent, those between 75th and 60th as very good, between 59th and 40th as average and below 40th as poor. Physical fitness performance was compared between gender.

### Breakfast consumption and its association with physical fitness and BMI z-score

Breakfast consumption was assessed by asking the school children if they consumed breakfast before coming to school on that day of the data collection. The questionnaire used was based on FAO’s nutrition knowledge, attitude and practices questionnaire for school children. One of the questions on the questionnaire was whether the child consumed breakfast that day. The response to this question was used to stratify the participants into breakfast consumers or non-consumers.

### Statistical analysis

All data collected were analysed using the Statistical Package for Social Sciences (Version 22) (SPSS) software (IBM Corp., 2013). All data variables were checked for normality using the Kolmogorov-Smirnov test, and examined whether they met parametric assumptions. The independent t-test was used for parametric data, while the Mann-Whitney ‘U’ test was performed on non parametric data. Descriptive statistics were conducted on demographic data collected. The Kruskal-Wallis test was run to determine the difference between individual physical fitness parameters across the weight groups, while partial correlation (controlled for age and gender) was used to determine the association between BMI z-score and MUAC measurement with the individual physical fitness scores. The former was done to test if fitness was related to the BMI z-score status of the children, while the latter was done to determine the direction and strength of associations between anthropometric and physical fitness scores. The associations between breakfast consumption and weight status, gender and fitness levels, and between exercise and fitness levels were determined using chi-square analysis for categorical variables or independent t-test for continuous variables.

## Results

### Prevalence of underweight and overweight

Mean age of the children was 11.1 ± 1.1 years. There was no significant difference (*p* = 0.942) between the mean age of boys (11.1 ± 1.1 years) and that of girls (11.1 ± 1.1 years). The sex specific anthropometric measurements of the pupils are summarized in Table 1. Mean BMI z-score was significantly higher in girls than in boys (*p* < 0.001), although the means put both groups in the normal BMI category. Likewise, the girls (22.0 ± 3.2 cm) had significantly (*p* = 0.021) higher mean MUAC compared to boys (20.7 ± 7.3 cm). The proportions of thin and overweight/obese children were 2.7 and 10.5% respectively (Table 2). Thinness was significantly higher in boys, more than 3 times than observed in girls, while the proportion of overweight or obese girls (14.6%), was more than twice that of overweight/obese boys (6.1%). Significant association was found between gender and BMI status among the pupils (x^2^ = 8.53, *p* = 0.003) (Table 2).

**Table 1:** Anthropometric characteristics of the study population

Measurement	Boys		Girls		Total		*P*-value
	Mean	SD	Mean	SD	Mean	SD	
Age (years)	11.1	1.1	11.1	1.1	11.1	1.1	0.942
Weight (Kg)	32.8	5.8	36.9	8.1	66.0	34.9	< 0.001
Height (cm)	140.5	7.0	144.2	8.4	163.2	142.4	< 0.001
BMI-for –age z-score	−0.43	0.9	−0.05	0.94	− 0.24	0.99	< 0.001
Height-for-age z-score	−0.51	1.0	−0.17	1.0	−0.33	1.1	0.001
MUAC (cm)	20.7	7.3	22.0	3.2	21.4	5.7	0.021

**Table 2:** Comparison of proportions of thinness, overweight and obese children by gender

			Total	Male	Female	Chi square	*P* value
BMI categories	Thin	Count	12	9	3	8.53	0.003
		%	2.7%	4.2%	1.3%		
	Normal BMI	Count	380	191	189		
		%	86.8	89.7%	84.0%		
	Overweight	Count	41	9	32		
		%	9.4%	4.2%	14.2%		
	Obese	Count	5	4	1		
		%	1.1%	1.9%	0.4%		

Chi square could not be used for the 4 BMI categories since some cells were small. To do that, the thin and normal were combined while the overweight and obese combined

### Physical fitness of the children

Table 3 contains a summary of the physical fitness performance. Apart from flexibility and handgrip, all other physical fitness tests achieved statistical significance between boys and girls. On the average, the girls scored higher in forward jump, 50 m run and overall fitness score, while the boys scored higher in sit ups. Comparison of mean total physical fitness score between in girls (20.1 ± 67.3) and boys (19.2 ± 8.3) suggests that the girls were overall fitter. However, on the whole, a larger proportion of boys (25.4%) performed excellently in overall physical fitness compared to girls (20%), but at the same time, more boys (43.2%) had poor overall fitness status compared to girls (38.7%) (Table 4).

**Table 3:** Physical fitness scores among school age children

Fitness test	Total		Boys		Girls		*P*-value
	Mean	SD	Mean	SD	Mean	SD	
Hand grip (kg)	4.4	2.2	4.1	2.4	4.5	1.9	0.069
Forward jump (cm)	4.6	2.1	4.1	2.2	5.0	1.8	< 0.001
Sit ups (s)	3.7	2.0	3.9	2.1	3.5	1.7	0.028
Flexibility (cm)	1.1	0.6	1.0	0.1	1.1	0.9	0.120
50 m run (s)	6.4	2.5	6.0	2.6	6.6	2.4	0.002
Total physical fitness score	20.1	7.3	19.2	8.3	21.0	6.2	0.012

Raw scores have been converted to standard scores ranging from one to ten for age and gender for physical fitness standards for Japanese children. *N* = 433

**Table 4:** Physical fitness category based on total physical activity standard score stratified by gender

Fitness category	Total n (%)	Boys n (%)	Girls n (%)	*P*-value n (%)
Excellent	99 (22.6)	54 (25.4)	45 (20.0)	0.019*
Very good	100 (22.8)	43 (20.2)	57 (25.3)	
Average	69 (15.8)	24 (11.2)	45 (20.0)	
Poor	170 (38.8)	92 (43.2)	78 (34.7)	
Total	438	213	225	

*denotes statistical significance. Standard scores refer to physical fitness standard cut-offs for Japanese children

### Relationship between physical fitness and BMI status

From Table 5, average handgrip score was significantly (*p* = 0.006) higher in overweight/obese children. All other physical fitness test scores did not show any significance with BMI status. Partial correlation between BMI z-score and physical fitness test scores, controlling for age, showed a weak positive significant correlation (*r* = 0.21, *p* < 0.0001) between BMI and hand grip, while a weak inverse correlation was observed between sit up score and BMI (*r* = − 0.113, *p* = 0.027). MUAC did not show significant correlations with any of the physical fitness measures.

**Table 5:** Comparing individual physical fitness scores by BMI status and their correlations

	Thinness	Normal	Overweight/obese	*P*-value	BMI z-score	
	Mean ± SD	Mean ± SD	Mean ± SD		Correlation	*P* value
Hand grip (kg)	3.0 ± 1.9^a^	4.3 ± 2.1^b^	5.0 ± 2.1^c^	0.006*	0.21	< 0.001
Forward jump (cm)	3.6 ± 2.4	4.6 ± 2.3	4.5 ± 1.7	0.235	0.06	0.199
Sit ups (s)	4.1 ± 2.0	3.7 ± 2.0	3.2 ± 1.9	0.105	−0.11	0.027
Flexibility (cm)	1.0 ± 0.2	1.1 ± 0.7	1.1 ± 0.4	0.235	−0.02	0.575
50 m run (s)	4.9 ± 2.7	6.5 ± 2.5	5.6 ± 2.3	0.088	−0.05	0.292
Total physical activity score	16.1 ± 8.5	20.3 ± 7.4	19.6 ± 5.9	0.445	0.03	0.565

Partial correlations are controlled for age. Correlations between fitness scores and MUAC were all not significant and not presented

### Breakfast consumption, and its association with weight status and and physical fitness

The proportion of pupils who consumed breakfast before school was 78.9%, while 21.1% did not consume breakfast before school (Table 6). No significant difference was found between boys and girls with regards to breakfast consumption (x^2^ = 0.366, *p* = 0.601) although breakfast consumption was slightly higher in boys (80.2%) than in girls (77.6%). Likewise, breakfast consumption was not associated with BMI status (x^2^ = 0.362, *p* = 0.865), that is, not different between thin, normal or overweight children. Some of the children (13.8%) indicated that consuming breakfast before school was difficult for them. Some of the reasons cited for the difficulties in consuming breakfast before school included; lack of breakfast at home due to lack of money or failure of mothers to prepare breakfast at home, and especially fear of being late for school. Other children indicated that tummy aches after breakfast consumption was a reason why breakfast consumption was difficult.

**Table 6:** Breakfast consumption by BMI-for age status, gender and age

			Thin	Normal	Overweight/obesity		Chi square	*P* value
Breakfast consumption	Yes	Count	7	236	33	276	0.359	0.549
		%	77.80%	78.40%	82.50%	78.90%		
	No	Count	2	65	7	74		
		%	22.20%	21.60%	17.50%	21.10%		
			Male	Female			0.366	0.601
	Yes	Count	134	142				
		%	80.20%	77.60%				
	No	Count	33	41				
		%	19.80%	22.40%				

*N* = 350. Breakfast consumption did not vary between different ages, not presented. Thin and normal children were combined for chi-square analysis between BMI status and breakfast consumption, since some cells were fewer than 5

None of physical fitness parameters varied between children who did and those who did consume breakfast, but for the fact that those who did not consumed breakfast performed better in 50 m run score than those who did (Table 7).

**Table 7:** Differences in average BMI z scores and physical fitness scores between breakfast consumers and non-consumers

Did you have breakfast before school	Yes	No	*P*-value
	Mean ± SD	Mean ± SD	
BMI z scores	−0.34C1.0	− 0.35 ± 1.1	0.800
HFA z-score	−0.28 ± 1.1	−0.24 ± 1.0	0.808
Hand grip (kg)	4.4 ± 2.2	4.6 ± 1.8	0.277
Forward jump (cm)	4.6 ± 2.1	5.0 ± 1.8	0.275
Sit-ups (s)	3.7 ± 2.0	4.0 ± 1.9	0.134
Flexibility (cm)	1.1 ± 0.8	1.0 ± 0.2	0.087
50 m run (s)	6.3 ± 2.5	7.0 ± 2.3	0.022*
Total physical activity score	20.0 ± 7.5	21.6 ± 6.4	0.423

*N* = 349

### Association between exercise and physical fitness

A high percentage (89.9%) of school children indicated that they engaged in some form of exercise, while 10.1% indicated that they did not do any exercise (*n* = 346). BMI z-score did not differ among children who reported engaging in exercise and those who did not (*p* = 0.364) but children who engaged in exercise scored an overall higher fitness score than those who did not (*p* = 0.021) (Table 8).

**Table 8:** Average BMI z scores and physical fitness scores between those who exercise and those who do not

	Do you exercise?		*P*-value
	Yes *n* = 311	No n = 35	
BMI z-scores	−0.357 ± 0.97	− 0.171 ± 1.15	0.364
Hand grip (kg)	4.5 ± 2.1	3.9 ± 2.3	0.148
Forward jump (cm)	4.7 ± 2.0	4.0 ± 2.0	0.070
Sit-ups (s)	3.9 ± 1.9	2.9 ± 1.8	0.003*
Flexibility (cm)	1.0 ± 0.3	1.5 ± 2.1	0.169
50 m run (s)	6.6 ± 2.4	5.1 ± 2	0.004*
Total physical fitness score	20.6 ± 7.2	17.5 ± 7.5	0.021*

*n* = 346

## Discussion

This study looked at nutritional status and physical fitness and their relationships with breakfast consumption among Ghanaian school-aged children. The children studied were from government-owned primary schools and over 85% of them were of normal BMI-for-age status. Most children from this socio-economic group are likely live active lives, such as walking to school, and engaging in various forms of physical activity both in and outside school. This contributes significantly to increased caloric expenditure, preventing positive energy balance and consequently obesity [14]. In this study also, less than 3% were thin or underweight, indicative that undernutrition is not significant though it exists. The level of thinness in the children studied was also much lower than the average prevalence of 35% reported for Africa [13], and suggests better nourishment in our sample. It should be noted that the children studied were in school and the schools were also all in an urban area. On the other hand, 10.5% of the children were at least overweight, of which 1.1% were obese. With the known consequences of overweight and obesity, such as cardiovascular diseases and type 2 [15, 16], there is the need for national surveys among school-aged children to confirm our findings, as this will warrant appropriate and timely interventions.

Underweight and overweight were both common in the children studied, although underweight was much lower, as 13.2% of the children were affected by under or overweight. The findings show that the double burden of malnutrition, reported among children below 5 years seems to perpetuate into the school age stage, and most likely will persist into adulthood. Our findings therefore confirm the growing concern of nutrition transition, with reports of high prevalence of childhood obesity in some developing countries [17, 18]. It is also more worrying that close to 2 in 10 of the girls, compared with 0.5 in 10 boys were at least overweight, suggesting that girls were at more risk of overweight and its consequences. Compared to boys of the same age, the growth spurt and maturity in girls occurs earlier during childhood and adolescence [19], and this could have contributed to the higher level of overweight in girls than boys. However, in Ghana, the overweight/obesity situation is higher among women and it is likely that this starts much earlier, when they are in the school age, as observed in this study. Overall, overweight appears to be a more significant challenge than underweight in this study, as it was also 4 times higher, and this implies that overweight is a greater challenge and can become a national burden in this age group if the trend continues [20].

Breakfast consumption was high among this sample, with over three quarters consuming breakfast the morning of the dietary assessment, although this was lower than reported in a previous study in Ghana, where 85.5% of school children had breakfast before school [21]. Moreover, fewer children reported a difficulty in consuming breakfast before school, suggesting that breakfast skipping was less than an issue in these children. Considered the most important meal of the day, lack of breakfast before school among school-aged children is associated with lack of concentration and consequently poor overall academic performance [22], and frequent breakfast skipping is a significant risk factor for the onset of metabolic syndrome among school-aged children [23]. Thus, the fewer proportion of children reporting difficulties in breakfast consumption observed is welcoming. In most primary and junior high schools within Ghana, stringent punctuality measures are in place and students may be punished for failure to comply. It is therefore not surprising that some children indicated fear of being late as a difficulty in breakfast consumption before school. This could mean that breakfast clubs in schools across the country would be of immense benefit with regards to improvement in breakfast consumption among school age children. Such initiatives have yielded impressive results in countries such as the UK and the USA. Frequent consumption of breakfast among school children has also been associated with healthy or lower BMI, as well as higher cardiorespiratory fitness in boys [24]. In this study, children who reported to consume breakfast and those who did not consume breakfast had similar BMI z scores, implying that the factors associated with overweight or thinness did not include breakfast consumption. Breakfast consumption should be promoted for the benefits mentioned above, and not necessarily to control body weight prevention.

Breakfast consumption was not related to any physical fitness level except for 50 m run. Some studies have reported the beneficial effect of breakfast in promoting physical and cardiometabolic fitness while others found no effect [25, 26]. In this study, the absence of a relationship between breakfast consumption and most physical fitness measures may be due to the fact that breakfast consumption or non-consumption was determined by one morning which was the morning of data collection.

With regards to physical fitness, close to 40% of the children performed poorly, and only a quarter were excellent. This should be looked at to ensure that these physically unfit children do not enter adulthood with it. The issue of increased physical inactivity among children remains a profound health challenge in most developed countries, and our findings show that this is also problem among children in developing countries, although the extent of physical inactivity may not be as that of the developed world. It is reported that physical activity accounts for only a small variation in the different measures of physical fitness among children [27], perhaps because reports of associations between fitness and physical activity among children populations have ranged from moderate to very weak [28]. Hence, although studies have shown that participation in exercise confers improved physical fitness in children, this association may not be necessarily causal. In our study, children who exercised were fitter that those who did not, confirming the benefit of physical activity on physical fitness. Girls have been showed to have lower levels of physical activity than boys [29–33] in contrast to what we found. It is worth noting that the scoring of physical fitness takes into account age and gender, and so the better performance by girls in this study actually indicates they are fitter. Differences in haematological markers and ventricular chamber sizes have been argued to account for variations in fitness levels among the sexes [34, 35]. However, the fact that more girls in our study reported taking part in physical activity than boys, and the girls were also fitter, suggest that their physical activity levels may be the reason for their fitness, and not the mentioned causes of differences between boy and girls.

Fitness performance has been showed to be negatively affected by excess weight gain in adolescents and children alike [36]. Several studies have reported higher physical fitness in normal weight children than overweight and obese children [29, 30, 37, 38]. In this study, overweight/obese children had the strongest handgrip while the thin had the least. Normal weight children in this study ran fastest, followed by overweight children, with the thin children trailing the list. A stronger hand grip indicates higher muscle mass [39] and conversely lower hand grip scores among thin children indicates lower muscle. Loss of muscle mass is common in malnourished children, a phenomenon which could be attributed to reductive adaptation in malnourished children. A study among a random sample of 3214 Flemish school children showed the obese participants had a greater handgrip strength than non-obese children [38], just like this study. For this study, overweight was assessed by BMI, which does not consider the composition of body weight [40]. It is likely therefore that the overweight children had more muscle mass, making them stronger. With the limitation of BMI in assessing muscle mass, future studies should assess body composition and not just BMI in order to ascertain whether stronger handgrip is associated with higher muscle mass. The thin children did more sit ups than the other groups but apart from that none of the fitness tests scores were different by BMI status. The inverse correlations observed between BMI z-scores and sit-ups was in line with that reported by Brunet et al.*,* (2007) [41] among Canadian school children while the significant positive correlations obtained between BMI z-scores and hand grips in this study is in coherence with a study among adolescents [42].

## Conclusion

In conclusion, insights have been provided into the breakfast consumption, physical activity practices, physical fitness and weight status and how these are interrelated in Ghanaian school-aged children in public schools. Findings suggest overweight is much higher in the children than thinness, while close to 40% had poor physical fitness status. While breakfast consumption was common in the sample, close to 15% were likely to skip breakfast. The double burden of malnutrition however exist in this school-aged children and can perpetuate into adulthood, but more studies are required to understand how dietary, physical fitness and BMI are related in school-aged children at the National level, in order to know where and how to intervene. Indeed, nutritional policies and interventions should not just target children under 5 years old and pregnant women, to the neglect of school-aged children, who are just a few years from being adolescents/adults.

### Limitations

In this study, the Japanese physical fitness standards for school-aged children were used to grade the performance of the Ghanaian children, due to lack of national standards for Ghanaian children. This was done to enable the conversion of raw physical activity scores to gender and age specific scores. Apart from using the standards to classify the children in this study, the children were not compared to Japanese children in terms of fitness, and and no interpretations have been made regarding whether Japanese or Ghanaian children are fitter. Also, significant limitations pertaining to self-reported measures of both physical activity [7] and dietary habits (46) especially among children have been reported. In this study, these data were collected by self-report and information given by these children could not be validated. Moreover, assessment of breakfast consumption was just for the day of data collection and this may not reflect the usual breakfast habits of these children. Finally, other factors that may influence breakfast consumption such as socioeconomic status and home environment were not included in this analysis. Because of the cross-sectional design of this study, causality cannot be inferred.

## Abbreviations

BMI: Body mass index
MUAC: Mid upper arm circumference
WHO: World Health Organisation
FAO: Food Agriculture Organisation
m: metres
cm: centimetres
Kg: kilograms
